# Recovery Agency and Informal Recovery Pathways from Gambling Problems

**DOI:** 10.1007/s11469-017-9747-x

**Published:** 2017-03-27

**Authors:** Sophie Vasiliadis, Anna Thomas

**Affiliations:** Australian Gambling Research Centre, Australian Institute of Family Studies, Level 20, 485 Latrobe Street, Melbourne, VIC 3000 Australia

**Keywords:** Informal recovery, Gambling, Agency, Self-help, Self-regulation

## Abstract

This study applied a holistic, strength-based lens to better articulate the impetus for, and processes of, informal recovery from gambling problems. Two research objectives framed the parameters of the study: to explore (a) the process by which gamblers move from recognition of a gambling problem to action for recovery and (b) the experiences, perceptions and contextual factors that shape the features of this process. Narrative telephone interviews were conducted with adult residents of Victoria, Australia. Thirty-two adult participants (22 males and 10 females) were recruited from the general community. All participants were self-identified as recovering or recovered from gambling problems. Participants primarily used informal recovery strategies, rather than professional services or support groups. The impetus for informal recovery was identified broadly as either (a) dissonance between desired and actual self-image and goals, (b) an uncontrollable adverse event, or (c) confrontation and decisive action by others affected by the individual’s gambling involvement. The impetus, process and goals of recovery were best described by pathways that were distinguished by agency in recovery: externally directed and self-directed. The application of a strength-based lens helped to illuminate the role of agency in informal recovery. A proposed pathways framework may inform strength-based informal recovery strategies for gamblers and affected others, and therapeutic approaches. The pathways, which have not been articulated in previous gambling recovery literature, generally cohere with pathways articulated in the alcohol and substance recovery literature.

In recent decades, the gambling industry has expanded into online platforms, and gambling products have become considerably more sophisticated and accessible (Australian Institute for Gambling Research 1999; Griffiths 2010; Productivity Commission 2010). International prevalence studies estimate that between 3 and 6% of adults satisfy criteria for moderate to severe gambling problems (Williams et al. 2012). An estimated 30–40% of these gamblers will recover using informal recovery processes, that is, rely on their own resources rather than professional or group support (e.g. Gamblers Anonymous) for recovery (Slutske 2006; Slutske et al. 2009). Researchers have argued that some of these individuals will recover “naturally”, “simply” mature out of gambling or gradually prioritise other activities (Anderson et al. 2009; Tonteatto et al. 2008). Informal recovery is achieved through the committed and intensive use of personal and social resources and strategies; however, the informal recovery process has not been fully articulated (Anderson et al. 2009; Granfield and Cloud 2001; Lubman et al. 2015; Moore et al. 2012; Tonteatto et al. 2008).

The scope of enquiry into informal recovery has generally been limited to identifying and quantifying strategies used, with limited attention to their context, or the impetus for their use as distinct from the recognition of a problem (Anderson et al. 2009; Hodgins and El-Guebaly 2000; Hodgins et al. 1999; Moore et al. 2012; Slutske 2006; Slutske et al. 2003, 2010; Tonteatto et al. 2008). Personal resources and contextual factors such as familial and peer relationships and employment opportunities have been suggested as moderators of the recovery process (McGowan 2003); however, whether they support or hinder, recovery has been described as “highly individualised and unpredictable” (Anderson et al. 2009 p. 45). A methodological limitation in the field has been that study participants have usually been recruited from professional services or community-based problem gambling support groups such as Gamblers Anonymous (GA), rather than the broader community (e.g. Hodgins 2001; Hodgins and El-Guebaly 2000; McGowan 2003; Moore et al. 2012; Slutske et al. 2010). Further articulation of temporal benchmarks, including impetus to recovery, as well as contextual factors and resource availability, would improve efficiency and effectiveness of resources to support people undertaking this recovery path.

A deficit perspective has dominated practice and discourse in the addictions (Ferentzy and Turner 2013; Keane 2000; Leung 2015; Neale et al. 2015). The perspective is based on assumptions that addiction is caused by dysfunction in the individual, is experienced as a chronic and debilitating disorder and that alleviation of the symptoms of the addiction are the overriding motivation for, and goal of, recovery. Gambling is no exception. For example, a consensus among a group of international experts in gambling research, self-named the Banff Consensus, proposed minimum standards for outcomes to be examined in treatment efficacy evaluations (Walker et al. 2006). The proposed outcomes pertain to gambling behaviour and gambling-related problems. One measure of positive outcome, quality of life, was considered an indirect outcome of recovery, and was suggested to compliment other measures. Evidence suggests, however, that informal recovery from gambling problems may not follow the proposed deficit trope of dysfunction, despair, and redemption through recovery (Anderson et al. 2009; Kimberley 2005; Neale et al. 2015; Tonteatto et al. 2008). Instead, some recovering gamblers have described their gambling as youthful recklessness, and recovery as a reappraisal of gambling and its role in their life, leading to a desire to revert to their “authentic self” prior to gambling problems (Anderson et al. 2009; Tonteatto et al. 2008).

This study therefore aimed to further articulate the informal recovery process in adult recovering gamblers by applying a holistic, strength-based lens. Two research objectives frame the parameters of the study to explore (a) the process by which gamblers move from recognition of a gambling problem to action for recovery and (b) the experiences, perceptions and contextual factors that shape the features of this process.

## Method

### Participants

The sample comprised 32 adults (22 males and 10 females) who had attempted informal recovery from a self-determined gambling problem. Participant scores on the Problem Gambling Severity Index (PGSI) (Ferris and Wynne 2001) ranged from 0 to 23, suggesting a broad range in current problem severity, and therefore recovery progress. In conjunction with informal recovery processes, 14 participants reported contact with gambling-related professional services. Apart from three participants who had received ongoing professional counselling or attended GA meetings, interactions were minimal, ranging from calls to a gambling helpline during gambling urges to registering with a gambling helpline but no further contact. A summary of demographic and gambling involvement information is provided in Table 1. Detailed methodological information has been previously published (Vasiliadis and Thomas 2016).

**Table 1:** Summary of demographic and gambling information of participants

Demographic characteristics	*n = 32*
Gender	
Male	22
Female	10
Age group	
18–24	4
25–30	10
40–49	7
50–59	3
60–69	7
70+	1
Employment status	
Full-time	13
Part-time	2
Casual	1
Self-employed	0
Retired	3
Pension	5
Unemployed	2
Volunteer	1
Relationship status	
Married/de facto	15
Single	10
In a relationship	6
Separated	1
Location	
Metropolitan	29
Regional	3
Gambling information	
Problem gambling severity	
Non-problem gambler	1
Low-risk gambler	3
Moderate-risk gambler	5
Problem gambler	23
Form of gambling most associated with problem	
EGMs	17
Racing	7
Casino table games	6
Online EGMs	1
Online casino table games	1

### Procedure

A convenience purposive sampling method was used, with participants primarily recruited through newspaper and social media advertisements. Two participants were recruited via contact information provided in a prior study conducted by SV. Recruitment of participants ceased when the full range of themes had been covered, and no new material was emerging (known as ‘saturation’) (Nixon and Wild 2008).

Participants were eligible if they (a) were aged 18 to 30 or 40+ years (to allow a comparison between older and younger participants, which was a research question of the larger study of which is paper is derived), (b) had experienced moderate to severe level gambling problems, (c) their recovery experience had been dominated by informal recovery processes, (d) their primary residence was in Victoria, Australia and (e) were able to verbally articulate in English their lived experience over the telephone. Degree of recovery progress was not a criterion; however, sampling emphasised variability in this aspect as we were interested in gaining insights across the full scope of the recovery process.

Ethics approval was granted by the Australian Institute of Family Studies Human Research Ethics Committee. Approval to re-contact eligible participants of a prior study was granted by the University of Melbourne Human Research Ethics Committee.

### Interviewing Schedule and Protocol

Telephone interviews ranged from 45 to 240 min were conducted by SV at mutually convenient times and were audio recorded and transcribed verbatim with participants’ consent. Speech disfluency (e.g. ‘ah’, ‘and the, the’) that did not add meaning to the participant’s narratives were not replicated here for clarity in reading. Unstructured narrative interviews were conducted (Jovchelovitch and Bauer 2000; Riessman 2008), whereby participants were instructed to ‘tell their story’, from gambling initiation to problem recognition and their response, through to the present day. Once the participant indicated that they had come to the end of their story, the interviewer probed according to the research questions to delve deeper into key events or processes raised by the participant (Jovchelovitch and Bauer 2000). Quantitative questions administered upon completion of the interview gathered information on demographics, service use and problem severity. For transparency, participant information regarding gender (M or F), age group and the primary gambling product/activity associated with the problem is provided with quotations.

### Data Analysis

An inductive-iterative approach was applied throughout data analysis across different forms of data, specifically transcriptions and interviewer notes. SV conducted all interviews and recorded narrative summaries and reflections following each interview. Interviews were transcribed verbatim by a professional transcription service. Applying narrative thematic analysis to the transcriptions, SV coded units of meaning into emergent themes using QSR NVivo10 software. Applying structural narrative analysis, SV then examined narrative arcs and features to identify temporal and dynamic relationships between major events, protagonists, bystanders, crises and conclusions (Jovchelovitch and Bauer 2000; Riessman 2008; Wolcott 2009). Further iterations of review were conducted whereby both authors reviewed, discussed and revised the findings. This process merged some minor themes into major themes, explored patterns within and across narratives and considered the implications of findings in terms of the research questions. Discussions between the authors and re-examination of raw data in relation to emerging themes throughout the analysis process ensured that the findings were grounded in the data (Berg 2001; Corbin and Strauss 2008). In the few instances where the adoption of understandings or nomenclature of professional therapies appeared to be evident in participants’ accounts, caution was taken in interpretation given the focus of the analysis was on informal recovery processes.

## Results

**Fig. 1:**
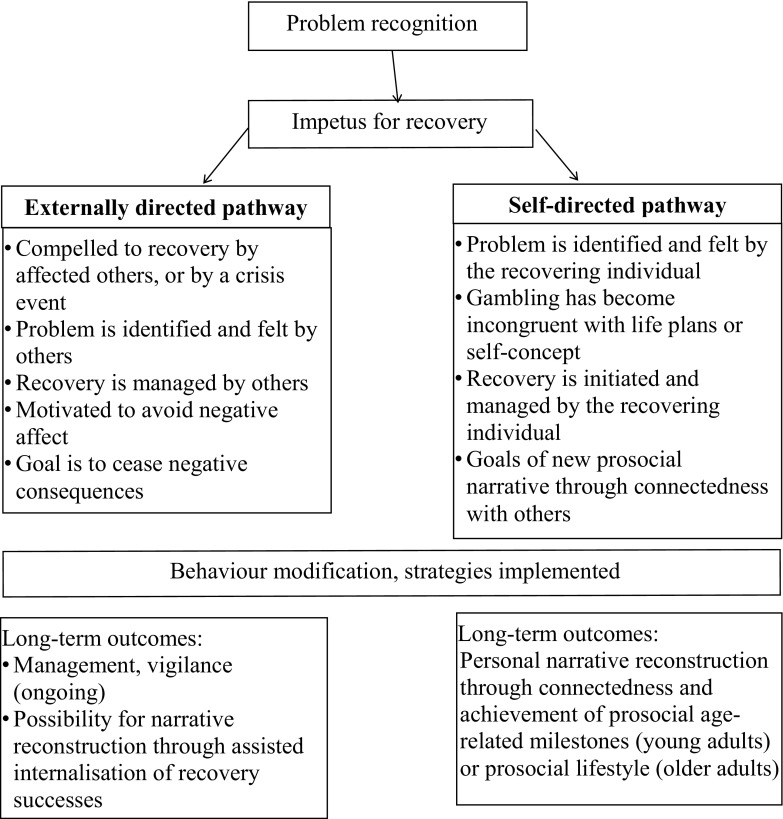
Externally directed and self-directed pathways of informal recovery from gambling problems

Emergent patterns in relation to recovery impetus, management and goals suggested informal recovery processes could be described by two recovery pathways, defined by the individual’s perception of agency in recovery (Pick et al. 2010). An externally directed recovery pathway describes participants who were motivated to recover through changed circumstances (e.g. reduced income, hitting rock bottom) or pressure from people around them (e.g. family, employer). Showing low personal agency, these participants required significant ongoing support and intervention throughout their recovery. A second self-directed recovery pathway describes participants whose recovery was motivated by the desire to achieve personal ambitions that had been inhibited by gambling (e.g. purchase a house, maintain a romantic relationship, raise a family, travel overseas). These participants showed personal agency in their self-managed recovery. Figure 1 summarises the key characteristics of the recovery pathways.

### Externally Directed Recovery Pathway

The narratives describing externally directed pathways positioned the gambler as the well-meaning but dishonest anti-hero, or the helpless fighter. As the participant quoted below explained, he liked to do ‘things the easy way and not wanting to work for it’ (M, 40–49, Casino table games). He and others who followed this pathway emphasised their struggle with ceasing gambling involvement on their own. The motivation to initiate recovery came from a desire for relief from negative consequences of gambling, such as poor mental health, financial hardship or relationship conflict. Some externally directed participants had recognised for some time that they had a problem with gambling but did not commence recovery until it was perceived as less of a struggle than the management of gambling consequences.

And whether it’s part of your character, you’re not strong enough, you’re weak or whatever it is, but some people are just powerless over stopping (M, 40–49, Casino table games)

The impetus for change for this group thus involved external forces that imposed change upon them involving (a) a significant negative change in circumstances or (b) the enforced interventionist approach of others. A significant negative change in circumstances usually involved hitting rock bottom financially and/or socially, or being caught out by significant others such as family members, friends or their employer. Being caught out was often precipitated by a sudden, uncontrollable event that simultaneously exposed the gambling problem and initiated a swift response from others. This response was often punitive in retaliation for harms incurred (e.g. a partner angry about significant household debt) or for a breach of social norms (e.g. friends admonish the gambler for gambling alone rather than socialising).

So when my friends found out and they rang my sister that was just beyond mortifying for me (F, 25–30, Online EGMs)

I got caught out because, like, coming home at 7.30 in the morning and everyone knew that I’d been at the casino … And there was a lot of people talking about my situation … it was quite overwhelming, the whole situation (M, 40–49, Casino table games).

An enforced intervention involved direct and decisive confrontation by significant others, whereby the gambler was held accountable for the consequences of their gambling. Significant others implemented and managed recovery strategies on behalf of the gambler on a regular, ongoing basis. This required considerable effort and resources, such as daily monitoring of all expenditure and movements. The participant below explained that the impetus and progress of recovery can be reliant on the intervention of others, in this case, his employer.

But until somebody sort of puts you aside and sits down with you at a table and sort of says, “Well, look we’re prepared to help you but you need to help yourself, because you do have a problem”, and you think, “Okay, maybe I do, yeah”. So you need somebody to sort of actually speak to you and say, “This is it, we’re prepared to do this for you if you do this and this and this”. And you think, “Okay, yes” (M, 60–69, EGMs).

Crucially, externally recovered participants required intensive, regular and ongoing assistance from others to implement and manage strategies for recovery. Family members usually adopted this role; specifically, this was the partner/spouse or adult child of older adult participants, or the partner/spouse or parents (if they were living with them) of young adults. This individual usually took responsibility for the management and monitoring of the recovering gambler’s finances to restrict their access to cash, and might monitor their activities, social interactions and exposure to gambling venues and products. Despite their reliance on intervention and accountability to others, externally directed participants still felt responsible for their recovery. An older woman expressed this sense of responsibility, ‘It is very difficult at times but I think at the end of the day it is up to me isn’t it?’ (F, 50–59, EGMs).

Importantly, in cases where the impetus was an enforced intervention, participants only embraced the recovery process when the agent of the impetus to change was respected and valued more highly than the perceived benefits of gambling. In the quote below, an externally directed participant shows his ambivalence towards recovery, as the concerns held by the agent (his wife) do not outweigh his enjoyment of the social environment at the gambling venue.

My wife was aware that I was spending too much time down there, was not happy about that particularly … So that set the scene for some conflict … Ah look, it it’s an awkward situation because I do have a wide group of friends and I also have a couple of my sons that we like to have a drink with and their mates [at the gaming venue] being younger and we all get on very well (M, 60–69, EGMs).

A few externally directed participants who had made some progress in their recovery indicated that they had now internalised efforts and successes in their recovery, even though they were clearly still heavily reliant on regular monitoring and support from others. These participants also indicated that they had recently started to redefine their self-image, had embraced a new role (e.g. grandparent, volunteer) or had started to think beyond present concerns and articulate personal goals and future aspirations. The quote below shows a participant’s transition from acknowledging her daughter’s intervention (i.e. a list of financial management strategies), to attributing those and other strategies to herself as she asks herself ‘what else did I do?’.

She [participant’s daughter] rang housing, and the gas and electricity [to arrange automatic payments]… And I really appreciate her doing that, because now my rent comes out fortnightly. I’ve got a car. I’ve paid so much for me car and me electricity and gas. Um, what else did I do? I pay my phone bill… Me sister-in-law takes me shopping. And I’m happy to go with her, because if I didn’t go shopping, I’d go to the pokies and waste me money (F, 60-69, EGMs).

Interestingly, she attributes to herself agentic qualities for going with her sister-in-law to the supermarket so that she can avoid local gaming venues. Internalisation of progress and the perception of personal agency might have been supported by the approach her daughter took, where she insisted her mother to use her own finances to restore financial stability. This seems to have helped the participant to feel that she had contributed to strategy implementation and recovery progress, and therefore provided a sense of agency.

### Self-Directed Recovery Pathway

Self-directed narratives positioned the gambler as the hero protagonist and primary agent of their recovery process. Participants whose narratives followed this pathway tended to have a strong sense of self-identity and clarity around their social roles and direction in life. They were instrumental in initiating and progressing their own recovery process and reconstructing their narrative in pursuit of personally identified and directed goals and ambitions.

Participants who followed the self-determined pathway framed recovery as the pursuit of prosocial lifestyles and achievements, such as starting a family, purchasing a property, improving career prospects or developing emotional maturity. High expenditure and preoccupation with gambling was seen as inhibiting their ability to achieve these ambitions. For example, a young man (M, 25–30, Casino table games) clearly articulated his recovery as a process of personal change and ‘a big, big transition period’ where he ‘stop[ped] being that sort of person’ when he met his future wife. An older man (M, 50–59, Racing) lamented ‘I should have gotten married years ago’ because, for him, his marriage represented the achievement of recovery, which also served to assist the ongoing maintenance of his recovery.

The ultimate recovery goal for these participants transcended relief from gambling consequences, to the construction of a new, positive and prosocial narrative. Young adults were more likely to express this type of narrative. They believed these achievements were important milestones for young adulthood, and they expected to achieve them. These ambitions were reinforced for those whose peers were achieving these milestones.

You know reaching that milestone in age, a lot of my friends are moving on with their lives, some are travelling overseas, working overseas, getting married, getting hitched, buying a home and I’m undecided, still like you know at the “Scene”, type of mentality. I took a step back and I said oh this can’t continue, yeah, I’m either going to end up broke or you know, or worse, who knows (M, 25–30, Casino table games).

The achievement of tangible milestones such as marriage and home ownership were more strongly associated with recovery for young men than young women, who by contrast more strongly associated recovery with personal development (e.g. improved coping strategies and emotional intelligence), connectedness with significant others and spiritual and philosophical engagement/meaning-making.

I’m taking a lot of Buddhist kind of thoughts on board like, well, can’t change the past and don’t blame anyone else for the situation that you’re in and sit down and work out what you want to do to be happy and then just do it (F, 25–30, EGMs).

Self-directed participants described high intensity gambling and problem severity, and asserted that they had an “addiction” to gambling as they prioritised it over other activities and responsibilities despite substantial or severe consequences. However, these participants also asserted that they had a choice in relation to gambling, which affirmed their self-determination and agency in recovery and provided motivation to commence and/or maintain recovery efforts. For example, one young man (M, 25–30, Casino table games) outlined gambling at a casino with friends and quickly falling ‘in love’ with roulette. He started gambling alone and more intensely (i.e. 6–9 h sessions four to five times/week). His health and wellbeing was affected through stress, anxiety, smoking, and poor diet. The experience of large wins and losses reduced his value of work which contradicted personal values of achievement through work and financial security. Recognition of these threats to his self-identity and ambitions motivated him to initiate recovery.

It’s up to you—you’ve got some sort of priorities and, you know, stop living this lie … I had to wake up and have to start making plans for the future (M, 25–30, Casino table games).

This meant making ‘mature decisions’. The cognitive process moved from a motivation phase (through recognition of consequences) to a volition phase of recovery strategy implementation. He understood his recovery as a process of personal development through the personal pursuit of future plans and financial goals. In recovery, he has stopped smoking, started competing in marathons, and is saving to purchase a house.

For some other participants, this realisation helped maintain commitment to the recovery process. For example, one young man (M, 18–24, Casino table games) ‘decided not to lie’ to his mother when she questioned him about his request to borrow money, instead telling her ‘I have got a gambling problem’. He also chose to be honest with his friends about how much he gambled following each session as a means of keeping himself accountable (he aimed to minimise, rather than cease, gambling). Thus, recovery was maintained through a series of ‘split second’ choices not to go to a gambling venue, or to be honest about expenditure. For these individuals, a sense of agency in recognising a choice to gamble and self-determination in recovery was essential to recovery.

Self-directed participants were also resourceful in accessing help from family and friends, but did not rely on others to guide and manage their recovery process. Their belief in their personal agency and pride in progressing the recovery process was one of the reasons this group also tended to opt against using formal services, and some against self-exclusion programs, as they perceived these as devaluing their own efforts not to gamble.

## Discussion

The study achieved its aim to further articulate the impetus for, and processes of, informal recovery from gambling problems. A strength-based approach to data analysis helped to articulate two informal recovery pathways, distinguished by agency in recovery: externally directed and self-directed. These findings are novel to the gambling field and have meaningful implications relating to informal recovery support.

Externally directed recovery narratives demonstrated limited personal agency in recovery. The impetus for recovery was externally driven, either through strong intervention from significant others or the experience of substantial negative consequences. The recovery process was only embraced when intensive practical support was available to sustain its ongoing management, making the recovery effort less challenging than the management of gambling problems. Participants’ primary goals in recovery focused on relief from harms through cessation of gambling.

Self-directed recovery narratives demonstrated personal agency through self-determination to achieve personal goals and milestones, which participants believed had been inhibited by high gambling intensity and preoccupation. Awareness of dissonance between desired and actual self-image and goals produced an ‘epiphany moment’ and provided the impetus for recovery. These participants initiated and managed their recovery through self-regulation and engaged the support of others if this was available. While also pursuant of relief from harms, their primary recovery objectives were a greater sense of meaning, purpose, and connectedness through the pursuit of prosocial life goals, constructive relationships and community involvement.

The findings generally cohere with alcohol and substance abuse recovery literature, which also recognises two pathways in informal recovery: avoidant-oriented and approach-oriented recovery (Granfield and Cloud 2001; Stall and Biernacki 1986; Walters 2000). This literature proposes a temporal relationship, where by informal recovery is initiated by the avoidance of gambling-related stimuli and harms, and maintenance of recovery is predominantly achieved with approach-oriented strategies, including involvement with family and the construction of a new narrative and roles (Stall and Biernacki 1986; Walters 2000). This temporality in the process was not observed in the present study; however, participants in both pathways used the strategies of diversion from gambling stimuli and social support. Further, in terms of externally directed gamblers, findings suggested positive reinforcement and internalisation of successes could assist them to develop personal agency and new narrative construction.

Interestingly, none of the participants of the present study expressed a desire to return to a former ‘authentic’ self, which has been described in previous gambling recovery literature (Reith and Dobbie 2012). Participants either did not discuss having an ideal lifestyle prior to gambling, or suggested that they were in a transition phase (e.g. emerging adulthood, retirement) that required that they construct a new narrative. In some cases, the implication was that to construct a healthy narrative and lifestyle in recovery, they must sever associations with their ‘gambling lifestyle’ as well as the lifestyle that preceded it. While self-directed participants had well-defined new narrative or identity aspirations, externally directed participants demonstrated less aspiration and tended to rely on others to guide their narrative or identity construction. Further exploration of how problematic gambling may dissolve or sever continuity of the self could better articulate the externally directed pathway and inform the development of targeted interventions and recovery support.

The present findings, together with those of alcohol and substance abuse recovery, suggest that recovery research and intervention viewed solely through a deficit perspective lens is insufficient, leading to incomplete and potentially inaccurate conclusions (McIntosh and McKeganey 2001; Pienaar et al. 2015). A number of narratives did not follow the recovery narrative structure of despair, destitution and redemption through recovery (Anderson et al. 2009; Kimberley 2005; Pienaar et al. 2015). Notably, the recovery goals of those following a self-directed recovery pathway transcended the relief from gambling consequences to positive future lives and possibilities around family, work and travel, concordant with their social circle. This finding was particularly relevant to young adult experiences and corresponded with findings from a study of young people in a 12-step substance abuse recovery program (Gonzales et al. 2012). These young people similarly did not identify with the deficit trope that structured their program. Substance abuse had significantly harmed many life domains; however, these young people believed they had agency in recovery, and viewed recovery as a process of constructing a new non-addict narrative around socially conventional personal achievements.

The current findings suggested that the integration of strength-based principles into recovery research and intervention could assist in shifting externally directed individuals to increase their sense of personal agency and ability to self-regulate to a state similar to self-directed individuals, and help prevent relapse (Petry 2005). Findings also highlighted the value of support networks promoting internalisation of recovery efforts and achievements through positive reinforcement and attribution to the recovering gambler. Although self-directed recovering gamblers have strong personal recovery agency, these individuals still accessed personal and social resources to fulfil their personal goals. This supports the provision of practical and relevant advice and support from professionals, programs and multimedia resources, and institutions (e.g. banks, employers, health services) for all recovering gamblers. This could take the form of assistance with resource access and management, advice regarding self-regulation strategies, and with provision of opportunities for enhancement of social integration and connectedness.

The important role of practical and emotional support from others and the resources required for narrative reconstruction are evident. Recovering individuals need assistance to develop their sense of self-identity and self-concept, and to envision and act on new life goals. Opportunities created by supportive networks can assist with the development of relationships and roles (e.g. spouse, grandparent, volunteer) that are meaningful and purposeful, and the articulation of goals and ambitions. These opportunities are considered essential to mental health and wellbeing, and recovery from alcohol and substance abuse (Best and Lubman 2012; McIntosh and McKeganey 2000).

These findings also point to a role for harm minimisation measures. Pre-commitment technology allows gamblers to set time and money limits prior to gambling, including to zero (Thomas et al. 2016). This technology can provide a physical barrier to gambling for externally directed individuals or enhance a sense of control and agency for self-directed individuals. This technology will only be effective for externally directed individuals where registration is a requirement to gamble; pre-set limits are irrevocable in the short term, and the program applies across a wide geographical or virtual region (e.g. apply to multiple online gambling websites). This technology can also provide gambling transaction history statements so that gamblers and their support networks can monitor changes in spending to enhance accountability and assist lapse prevention.

Limitations in the use of narratives must be acknowledged. Narratives are subjective stories that are constructed and reconstructed by individuals over time and in response to the interests of their audience (Jovchelovitch and Bauer 2000; Riessman 2008). While this subjectivity provides interesting insight into participant perspectives, triangulation of information sources is important to address concerns of validity (Denzin and Lincoln 2011). At times during interviews, some participants appeared to be unaware of, or wished to downplay, the severity of the impact of their gambling on others. Given the importance of significant others shown in this research, future research that collected the experiences of people impacted by, and supporting of, the recovering gambler in addition to those of the gambler would provide a holistic understanding of the experience.

This was a small-scale exploratory study. A larger multi-method study is now required to examine and further articulate the validity and generalisability of the proposed recovery pathways. In particular, research is required to examine whether the pathways apply equally across important subgroups relating to gender, problematic gambling activity and geographic location. Additionally, a prospective methodology would better examine the long-term effectiveness of recovery approaches and examine the cycles of recovery and relapse. More specifically, this method could examine the process by which externally directed individuals may develop a self-directed recovery approach and the risk of relapse for these individuals relative to other externally directed participants (Laudet et al. 2002).

In recent years, many fields have been moving towards more holistic and strength-based approaches. The present findings contribute to the growing body of recovery literature that references the usefulness of positive psychology and mindfulness (Krentzman 2013) and support the merits of the strength-based approach (Cederbaum and Klusaritz 2009). While these principles have begun to be adopted in problem gambling clinical practice (de Lisle et al. 2012; Toneatto et al. 2007), their role in vulnerability to a problem and recovery have received a relatively little attention in gambling research.

## Conclusion

The application of a holistic, strength-based lens to the informal recovery process from gambling problems helped to further articulate the impetus for, and processes of, recovery from gambling problems. A new framework is proposed to understand and support informal gambling recovery processes by adults. The framework is oriented on the individual’s personal recovery agency, that is, whether the participant positioned themselves (self-directed), or others or circumstances (externally directed), as the driving agent of the initiation and management of the recovery process. The recovery goal of externally directed individuals was for relief from negative gambling consequences, while self-directed individuals aimed for a positive, non-gambling narrative, including new roles, and meaning through connectedness. These pathways propose a strength-based heuristic with which to understand and support informal and formal recovery processes. These recovery pathways, which have not been articulated in previous gambling recovery literature, generally cohere with pathways articulated in the alcohol and substance recovery literature.
